# Utilization and Cost of Health Services in Individuals With Traumatic Brain Injury

**DOI:** 10.5539/gjhs.v7n6p156

**Published:** 2015-04-15

**Authors:** Clara E. Dismuke, Rebekah J. Walker, Leonard E. Egede

**Affiliations:** 1 Health Equity and Rural Outreach Innovation Center (HEROIC), Charleston VA HSR&D COIN, Ralph H. Johnson VAMC, Charleston, SC, USA; 2 Center for Health Disparities Research, Medical University of South Carolina, Charleston, SC, USA; 3 Division of General Internal Medicine and Geriatrics, Department of Medicine, Medical University of South Carolina, Charleston, SC, USA

**Keywords:** traumatic brain injury, utilization, cost, mental health

## Abstract

Traumatic Brain Injury (TBI) has gained attention in the past decade as a “signature injury” in the conflicts in Iraq and Afghanistan. TBI is a major burden for both the military and civilian population in the US and worldwide. It is a leading cause of death and disability in the US and a major health services resource burden. We seek to answer two questions. What is the evidence regarding the association of TBI with health services utilization and costs in the US and worldwide? What is the evidence regarding racial/ethnic, gender, geographic, socio-economic and other disparities in health services utilization and cost in the US and worldwide? To attain this goal we searched several databases using key words to perform a systematic review of the literature since 2000. We found 36 articles to be eligible for inclusion in the review. The evidence demonstrates a wide variation in health services utilization and costs depending on population of study and severity of TBI. The evidence also supports the existence of racial/ethnic, gender, insurance, geographic disparities in the US as well as other unique disparities worldwide.

## 1. Introduction

Traumatic Brain Injury (TBI) has gained attention in the past decade as a “signature injury” in the conflicts in Iraq and Afghanistan (Egede, Dismuke, Echols, 2012). TBI is defined as a physical force to the brain sufficient to cause structural alteration or physiological disruption of brain function that results in altered consciousness, amnesia, change in mental state, neurological deficits, or intracranial lesions. TBI is classified by severity as mild, moderate or severe. Mild TBI is the most common type among veterans who sustain a TBI (Morgan et al., 2012). Approximately 313,816 military service members have been diagnosed with TBI since 2000 (Defense and Veterans Brain Injury Center, 2015). However, TBI is also a major health burden in the US civilian population with approximately 1.7 million TBI injuries annually (Faul et al., 2010). Moreover, TBI is a leading cause of death and disability in the US with approximately 53,000 persons (18.4 per 100,000) dying from TBI related injuries annually (Coronado, Xu, Basavaraju et al., 2011). Fire-arms (34.8%), motor-vehicle accidents (31.4%) and falls (16.7%) are the leading causes of TBI related death (4).

TBI is a major health services resource burden with approximately 1,365,000 (80.7%) emergency department (ED) visits and 275,000 (16.3%) hospitalizations annually (Faul et al., 2010). More recently, evidence has shown that TBI may be accompanied by mental health co-morbidities in the military population, especially PTSD (Taylor et al., 2012). Given the large health and resource burden of TBI, we conducted a systematic review of the literature on health services utilization and costs associated with TBI. We sought to answer the following questions: 1) What is the evidence for TBI associated health services cost and utilization in the US and worldwide? 2) What is the evidence for racial/ethnic, gender, geographic, socio-economic and other disparities in health services cost and utilization in the US and worldwide? We were specifically interested in post-injury cost, rather than initial hospitalization cost.

## 2. Method

### 2.1 Information Sources, Eligibility Criteria and Search

In order to answer these two questions, three databases (Medline, PsychInfo, and CINAHL) were searched for articles published between January 2000 through June 2013 using a reproducible strategy. Four searches with broad search terms were performed in each database using MeSH headings search. The first search used the terms *traumatic brain injury* and *cost*, the second used *traumatic brain injury* and *utilization*, the third used *traumatic brain injury* and *outpatient*, and the fourth used *traumatic brain injury* and *ambulatory care*.

The following inclusion criteria were used to determine eligible study characteristics: (1) must be published in English, (2) must include health services cost or utilization related to TBI (3) must include adults. Exclusion criteria included: (1) did not focus on clinical efficacy, (2) was not limited to describing cost-effectiveness of an intervention, and (3) did not focus on pediatric TBI.

### 2.2 Study Selection and Data Collection

**Figure 1:**
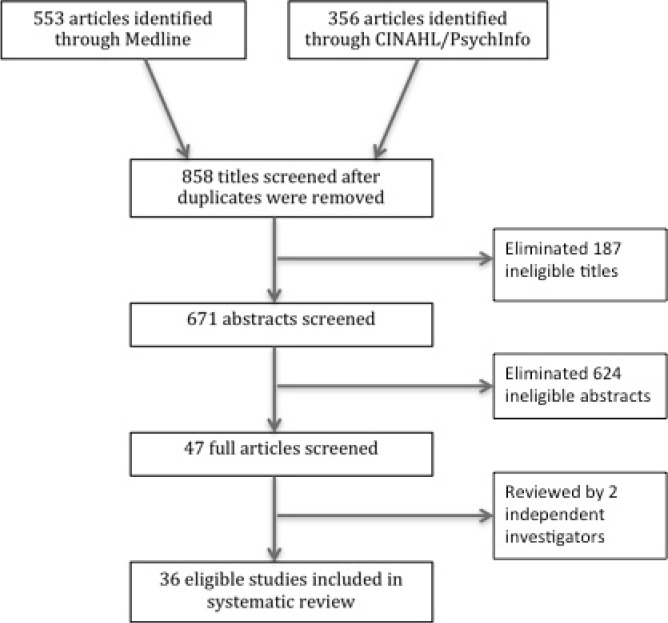
Process for eligible article selection

The process used to screen the citations is shown in Figure 1. Titles were eliminated if they were obviously ineligible, for instance describing cost-effectiveness of an intervention or including children with TBI. Full articles were read and reviewed using a standardized check-list by two independent reviewers (CD, RW). A third independent reviewer (LE) was asked to make the final decision regarding eligibility in the case of disagreement.

Data collected from the eligible articles is shown in Tables 1, 2 and 3. A summary of the evidence in each article is presented specific to health services costs (Table 1), health services utilization (Table 2) and disparities in cost and utilization (Table 3). We separated our review and tables by disparities due to our findings of a number of articles encountering racial/ethnic, geographic and other socio-economic disparities in the US and around the world. A narrative review was performed because of the heterogeneous nature of the information, which precluded conducting a meta-analysis.

**Table 1:** Cost studies

Study Title	Study Author(s)/Year	Findings	Type of Cost
Prevalence and Costs of Co-occurring Traumatic Brain Injury With and Without Psychiatric Disturbance and Pain Among Afghanistan and Iraq War Veteran VA Users.	Taylor et al., 2012	OEF/OIF Veterans with TBI in 2009 had 4 times higher median cost relative to those without TBI. Those with TBI and PTSD had higher median cost. The median annual cost per OEF/OIF Veteran diagnosed with TBI was $5,831 as compared to $1,547 for Veterans without TBI. Among Veterans with TBI, the median annual cost for those with TBI, PTSD and pain was $7,974.	Total VA health services costs for OEF/OIF Veterans with TBI, with and without comorbid PTSD.
Non-surgical Intervention and Cost for Mild Traumatic Brain Injury: Results of the WHO Collaborating Centre Task Force on Mild Traumatic Brain Injury	Borg et al., 2004	Review article of non-surgical intervention and mild TBI costs. Found indirect costs are much higher than direct medical costs. Admission and radiological policies are determining factors of direct costs.	Direct and Indirect Costs For Mild TBI.
Trend and Geographic Analysis for Traumatic Brain Injury Mortality and Cost Based on Market Scan Database	Hu et al., 2013	For 52,721 privately insured and 23,592 Medicare insured patients with TBI from 2004 to 2009, median hospital costs for adults were $13,000 for 18-64 and $9,000 for 65+. Significant differences in cost by geographic region were also found with the highest costs in California and Washington.	Privately insured and Medicare inpatient hospital costs.
Utilization and Costs of Health Care after Geriatric Traumatic Brain Injury	Thompson et al., 2012	Among adults aged 55-84 years old in the National Study on the Costs and Outcomes (NSCOT) database diagnosed with TBI, unadjusted mean costs ranged from $72,733 in the 75-84 group to $77,872 in the 55-64 group. In adjusted cost model, index hospitalization and inpatient rehabilitation costs were lowest among 75-84 while outpatient care and nursing home costs were lowest among younger ages.	Index hospitalization, inpatient, outpatient and nursing home costs for geriatric TBI.
The Direct Economic Burden of Blunt and Penetrating Trauma in a Managed Care Population	Davis KL et al., 2007	Initial hospitalization charges ranged from $32,627 for TBI alone to $103,667 for TBI along with other trauma. Initial hospitalization charges were highest for those admitted to Level 1 trauma centers except for TBI with other trauma while charges were highest for nontrauma centers.	Initial hospitalization charges for TBI with and without other trauma.
Mental Illness, Traumatic Brain Injury, and Medicaid Expenditures	Wei et al., 2005	Among a Medicaid population in four states, the presence of comorbid mental illness increased the cost of care for those with TBI. For those without severe mental illness (SMI) and TBI, total expenditures were $6,093 compared to $8,723 for those with TBI and an SMI in Alabama, $$6,919 vs. $13,688 for Georgia, $21,924 vs. $10,907 for New Jersey, and $7,154 vs. $19,986 for Wisconsin.	Medicaid cost for TBI with and without comorbid mental illness.
Outcomes and costs of acute treatment of traumatic brain injury	McGarry L et al., 2002	For patients aged 16 and older hospitalized for TBI between 1/1/97 and 6/20/1999, costs of hospitalization ranged from an average of $8,189 for moderate to $33,537 for critical TBI. Costs ranged from $15,860 for falls to $20,084 for gunshot wounds and 20,522 for motor vehicle accidents.	TBI inpatient hospital cost for moderate and critical TBI.
Charges and lengths of stay for acute and inpatient rehabilitation treatment of traumatic brain injury 1990-1996	Kreutzer et al., 2001	Over a 7 year period between 1990-1996, acute care daily charges showed almost routine increases while lengths of stay generally showed a downward trend with annual reductions averaging 2.25 days. The rise in rehabilitation charges was offset by corresponding decreases in lengths of stay. Increases in daily charges for rehabilitation were comparable to general medical care prices while rate of change in acute care charges was substantially greater, about 10% higher than medical care prices.	Length of stay utilization and charges for inpatient hospital and inpatient rehabilaition for TBI.
The Costs of Traumatic Brain Injury	Thompson K, 2001	Mean cost of treating a TBI at TBI Model System facilities were about $98,612 per case while the mean costs of inpatient rehabilitation were $43,212, not including physician charges.	Inpatient hospitalization and inpatient rehabilaition cost at TBI Model System Rehabiliation facilities for TBI.
Health Care Costs Associated with Traumatic Brain Injury and Psychiatric Illness in Adults	Rockhill et al., 2011	Average costs were 76% higher in the 3 years after injury for the mild TBI group and 5.75 times greater for moderate to severe TBI group when compared to controls. Presence of psychiatric illness associated with more than doubling of total costs for both inpatient and outpatient non-mental health care.	Inpatient and outpatient costs of TBI with and without comorbid psychiatric conditions.
Medical Care Costs Associated with Traumatic Brain Injury over the Full Spectrum of Disease: A Controlled Population-Based Study	Leibson et al., 2012	For incident TBI in the Rochester Epidemiology Project (REP) among residents of Olmsted Minnesota, most incremental costs occurred within the first 6 months while significant long-term incremental costs were not apparent among 1 year survivors. Cost differences between possible TBI cases and matched controls were not as great in the first 6 months, but were substantial among 1 year survivors.	Short and long-term incremental costs for 1 year survivors of TBI.
Health and Economic Burden of Traumatic Brain Injury: Missouri, 2001-2005	Kayani et al., 2009	During 2001-2005, mean cost per hospitalization and ED visit was approximately $6,948..	Inpatient hospitalization and emergency department costs for TBI.
The contribution of traumatic brain injury to the medical and economic outcomes of motor vehicle-related injuries in Ohio	Rochette LM et al., 2009	Among injured roadway users in Ohio between 2003 and 2006, when compared with a non-TBI injury, total hospital charges increased by a factor of 1.35 for a TBI. Mean hospital charges were $46,441 for a TBI roadway injury versus $32,614 for a non-TBI roadway injury.	Inpatient hospital charges for TBI and non-TBI roadway injuries.
Long-Term Medical Care Utilization and Costs Among Traumatic Brain Injury Survivors	Vangel SJ et al., 2005	Among 63 participants in Michigan, residential, home health and state case management billings accounted for 15% of total billing, nearly half of total charges and 27% of Medicaid payments. Medications accounted for 39% of total billings, 19% of Medicaid payments and 7% of charges. Outpatient services (excluding primary care program services) contributed 21% of total billings, 24% of total charges and 31% of Medicaid payments. Primary care accounted for 13% of billings, 4% of charges and 6% of Medicaid payments. Emergency room visits contributed 12% of billings, 8% of charges and 7% of Medicaid payments. Motor deficits (FIM score) at discharge from inpatient rehabilitation showed inverse relationship to billings	Long-term care costs for TBI.
Characteristics of acute treatment costs of traumatic brain injury in Eastern China-a mulit-centre prospective observational study	Yuan et al., 2012	Hospitalization costs were highest for traffic accidents and lowers for blows to the head. In adjusted analysis, lower Glasgow coma score, longer LOS, male sex, transient patient status. Traffic accident, injury occurring on a construction site, treatment at a tertiary hospital, neurosurgical ICU, ICU stay, polytrauma and those needing neurosurgery had significantly higher costs while good recovery and self-paying patients had lower costs. A double LOS was associated with a 1.6 times higher hospital cost.	Inpatient hospital costs of TBI in China.
The economic cost of brain disorders in Europe	Olesen et al., 2012	The latest estimate for the cost of TBI in Europe in 2010 was €2,697 in direct health care costs.	Direct costs of TBI in Europe.
Determinants of hospital costs associated with traumatic brain injury in England and Wales	Morris S et al., 2008	Mean costs per patient were highest in those with an Abbreviated Injury Scale (AIS) of 5. Length of stay in critical care accounted for 51% of mean total costs, regular ward 38% and travel costs 5%. Those aged 45-64 years had highest costs. Costs were highest among those injured in motor vehicle accidents, those with a lower Glascow coma score (GCS) and those with a higher AIS. Co-existing neck, thorax, abdomen and spine also increased costs. Surgery was associated with higher costs as was being seen in a hospital with a neurosurgical unit, being seen by a specialist from anesthesia, orthopedics, and general surgery. Mortality was associated with lower costs.	Inpatient hospital costs of TBI in England and Wales.
Economic evidence in trauma: a review	Berg J, 2004	Based on 2004 data, average inpatient cost for TBI in Germany was €2,529, compared with €2,833 in Spain and €3,024 in Sweden. Average inpatient cost for concussion was €1,071 for Germany, €987 for Spain, and €927 for Sweden. Average inpatient cost for severe brain injury was €6,647 for Germany, €6,362 for Spain and €6,045 for Sweden.	Inpatient hospital cost for TBI in Germany, Spain and Sweden.

**Table 2:** Utilization studies

Study Title	Study Author(s)/Year	Findings	Type of Utilization
Traumatic Brain Injury in the United States: Emergency Department Visits, Hospitalizations and Deaths, 2002-2006	Faul et al., 2010	TBI injuries were associated with 275,000 hospitalizations and 1,365 million ED visits in the US.	Inpatient hospital and emergency department utilization in TBI.
Pharmacotherapy Regimens Among Patients with Posttraumatic Stress Disorder and Mild Traumatic Brain Injury.	Morgan et al., 2012	In a study of OEF/OIF Veterans with co-morbid TBI and PTSD compared with with PTSD alone, the patients with PTSD and TBI were more likely to be prescribed an antidepressant a sedative-hypnotic, or an antipsychotic. The patients with TBI were also significantly more likely to receive psychotropic polypharmacy and to receive higher doses of psychiatric medications.	Pharmaceutical utilization in OEF/OIF Veterans with TBI and comorbid PTSD and PTSD alone.
Outcomes and Costs of Acute Treatment of Traumatic Brain Injury	McGarry et al., 2002	More than 4/5 of study subjects received CAT scan while MRI and electroencephalography were used less frequently. Use of all diagnostic tests increased with Abbreviated Injury Scale (AIS) score. Surgery and mean days on vent increased with AIS. 1/5 with critical TBI received ventriculostomy Most frequently used medicines were analgesics and anxiolytics as well as anticonvulsants in more severe injuries. 1/3 were on mechanical ventilation and 2/3 received ICU care.	Imaging, surgery, ICU and ventilator utilization in TBI.
The contribution of traumatic brain injury to the medical and economic outcomes of motor vehicle-related injuries in Ohio	Rochette LM et al., 2009	Among injured roadway users in Ohio between 2003 and 2006, when compared with a non-TBI roadway injury, the odds of requiring a ventilator (OR=3.66) and being admitted to the ICU (OR=2.51) were significantly higher for a TBI roadway injury.	Ventilator and ICU utilization for roadway injuries with and without TBI.
Long-Term Medical Care Utilization and Costs Among Traumatic Brain Injury Survivors	Vangel SJ et al., 2005	Most frequent prescriptions filled were anticonvulsant/mood stabilizers.	Prescription utilization in TBI.
Economic evidence in trauma: a review	Berg J, 2004	CT utilization in Spain is much lower than Sweden and German with only 6% use.	CT utilization in Spain, Sweden and Germany.
Emergency department management of mild traumatic brain injury in the USA	Bazarian et al., 2005	Of the patients with isolated mild TBI, 44.3% underwent computed tomography, 23.9% underwent other non-extremity, non-chest x rays, 17.1% received wound care and 14.1% received intravenous fluids. However, only 43.8% had an assessment of pain. Of those with documented pain, only 45.5% received analgesics in the ED. Nearly 38% were discharged without recommendations for specific follow up.	Imaging, wound care, IV and pharmaceutical utilization in mild TBI.
Service Utilization following Traumatic Brain Injury	Hodgkinson et al., 2000	81% of sample used medical and allied health services and 66% used transport, and 40% used vocational rehabilitation services.	Medical, allied health, transportation and vocational rehabilitation utilization in TBI.
A multi-Center Analysis of Rehospitalizations Five Years after Brain Injury	Marwitz JH, et al., 2001	Rehospitalization rates among a sample of patients with TBI declined between the one and five year follow up period from 23% to 17%. During the first year orthopedic and reconstructive surgery (25%) were the primary reason, dropping to 13% in year 5. Infections accounted for 10% of readmissions at 1 year follow-up and 8% at 5 years after injury. Pneumonia was the most frequent infection (44%) at 1 year and 5 years (43%). Incidence of rehospitalizations relating to seizures ad psychiatric disorders were 12% at one year and rose to 19% at 5 years.	Rehospitalizations in TBI.
Underutilization of neuropscychology in traumatic brain injury rehabilitation: Is managed care to blame?	Schatz et al., 2001	Only 26% of 273 patients with TBI received a neuropsychology evaluation (NPE). They were younger, more likely to be involved in liability claims, achieved a higher functional ability in primary rehabilitation and attended multiple rehabilitation facilities.	Neuropsychology evaluation utilization in TBI.
Severity of injury and Service Utilization Following Traumatic Brain Injury The First 3 Months	Philips VL et al., 2004	Among 113 individuals treated in Georgia TBI Model Systems, during first 3 months post rehabilitation discharges, at least 80% saw an MD, 42% reported 4 or more MD visits, more than 50% attended day rehabilitation programs, 42% had physical therapy, 36% occupational therapy, and 33% speech pathology. 11% psychological counseling.	Physician, rehabilaition and mental health utilization in TBI.
Rehabilitation of traumatic brain injury in Italy: a multi-center study.	Zampolini et al., 2011	Mean LOS was 87.31, 40.4% of patients had access to rehabilitation facilities after a month.	Length of stay and rehabilaition utilization in TBI in Italy.

**Table 3:** Disparities studies

Study Title	Study Author(s)/Year	Findings	Type of Utilization/Cost
Impact of socioethnic factors on outcomes following traumatic brain injury.	Heffernan et al., 2011	In a Level 1 trauma ED population, private insurance associated with shorter length of stay and intoxication with longer length of stay. Non-Caucasian race and lack of insurance associated with lower likelihood of placement in rehabilitation.	Length of stay, emergency department and rehabilaition utilization in TBI.
The effect of insurance status, race, and gender on ED disposition of persons with traumatic brain injury.	Selassie et al., 2004	In a state of SC hospital ED population, after adjusting for demographic, clinical and hospital characteristics, uninsured and black females had lower likelihood of hospitalization	Emergency department and hospitalization utilization in TBI.
Ethnic and racial disparities in emergency department care for mild traumatic brain injury	Bazarian et al., 2003	In a national sample of ED visits, after controlling for confounders, Hispanics more likely to receive nasogastric tube, nonwhites more likely to receive care by a resident and less likely to be sent back to a referring physician after ED discharge	Nasogastric tube and physician utilization in TBI.
Does Health Care Insurance Affect Outcomes After Traumatic Brain Injury?	Alban et al., 2010	Insured patients had longer ICU stay in unadjusted analysis.	ICU utilization in TBI.
Traumatic Brain Injury Hospitalizations Among American Indians/Alaska Natives	Rutland-Brown W. et al., 2005	Among 182,130 individuals hospitalized with TBI in 13 states, American Indians and Alaskan Natives (AI/AN) had the highest age adjusted rates of hospitalization for TBI relative to other race/ethnicities. The greatest difference occurred for individuals between 20 and 44 years old. High blood alcohol levels and low use of vehicle restraints were more prevalent in AI/AN.	Hospital utilization in TBI.
Measuring Unmet Needs and Services Among Persons with Traumatic Brain Injury	Heinemann et al., 2002	Most prevalent unmet needs were for memory (50.5%) job skills (46.3%), increasing income (50.5%). Black, younger and single individuals as well as those dependent in one or more daily activities and with more recent injuries had greater unmet needs.	Unmet Needs in TBI
The Use of Medicaid Waivers and their Impact on Services	Spearman RC et al., 2001	A review of TBI Medicaid Waivers in six states allowing flexibility in care and design of integrated service plans for those with TBI showed that the ability to obtain a waiver was associated with social-medical-political climate, similarity to other waivers, ability to strengthen access and reduce barriers, and expenditure of resources. Managing waivers was associated with cost effectiveness, developmental process of waiver implementation, ability to improve access and reduce barriers, and expenditure of resources.	Medicaid Waiver Utilization in TBI.
National estimates of hospitalization charges for the acute care of traumatic brain injuries	Schootman M. et al., 2003	Mean and median acute care charges were only slightly higher for males compared with females however the median age increased with age until age 34 after which it remained stable. Mean and median charge increased with increasing injury severity. Mean and median charges were highest for the western region of the US and for persons covered by Medicaid and those treated at urban teaching hospitals.	Inpatient hospital charges in TBI.
Is There Equity in Long-Term Healthcare Utilization After Traumatic Brain Injury?	Willemse-van Son MA 2009	Among 79 patients with moderate/severe TBI in Netherlands, those with a high locus of control with the physician were more likely to visit medical specialists and use supportive care than those with lower levels of internal locus of control.	Physician and supportive care utilization in TBI in Netherlands.
Sixteen years on: Has quality of care for rural and non-compensable traumatic brain injury clients improved	O’Callaghan et al., 2009	Rural TBI patients more likely to be treated in non-inpatient setting than urban TBI patients in Australia though no significant difference in functional outcome.	Non-inpatient utilization in TBI in Australia.
Rehabilitation Outcomes of Terror Victims with Multiple Traumas	Schwartz et al., 2008	Terror victims with TBI had higher rates of brain surgery but no difference in length of stay in hospital relative to non-terror victims in Israel.	Surgical utilization in TBI in Israel.

## 3. Results

### 3.1 Study Selection

Of the 858 articles found through searching three databases, 36 were eligible for inclusion in this review. See Table 1 for process details. Fourteen articles provided information on cost of TBI alone (Table 1), seven provided information on utilization in TBI alone (Table 2), four provided information on both cost and utilization (Tables 1 and 2) and eleven provided information on disparities in cost and utilization (Table 3).

### 3.2 Cost and Utilization Analysis

Type of cost and utilization are included in Tables 1 through 3 for each paper reviewed. Cost referred to monetary value either measured in real cost or charges when real cost was not available. Cost included both direct and indirect cost. Monetary value was measured in dollars for the US and the appropriate currency for international studies. Variability in cost can be due to provider differences, third party payer differences, and country differences. For this reason, utilization is a much better standard for comparing resource use, especially between health systems and countries.

## 4. Discussion

### 4.1 Health Services Cost Associated With TBI in the United States

Costs associated with TBI vary widely depending on the population studied, severity of injury and time period. In an early review of mild TBI without surgical intervention, indirect costs were much higher than direct costs, accounting for 92% of total costs in 1981 dollars (Borg et al., 2004). Admission and radiologic policies were found to be determining factors in the level of direct costs (Borg et al., 2007). A study conducted between 1997 and 1999 found that costs of hospitalization ranged from an average of $8,189 for moderate to $33,537 for critical TBI (McGarry et al., 2002). Those due to falls averaged $15,860, while those due to gunshot wounds averaged $20,084 and motor vehicle accidents averaged $20,522 (McGarry et al., 2002). In a study from 1990-1996, acute care daily charges showed routine increases, while lengths of stay generally decreased. (Kreutzer et al., 2001) Rehabilitation charges were about 10% higher than medical care prices, and offset the corresponding decreases in lengths of stay. (Kreutzer et al., 2001)

Since 2000, there have been a number of studies in the US and world wide of health services costs associated with TBI in various populations with costs ranging from $9,000 to $103,667. (Borg et al., 2004; Hu et al., 2013; Thompson et al., 2012; Davis et al., 2007; Wei et al., 2005; McGarry et al., 2002; Kreutzer et al., 2001; Thompson, 2001; Rockhill et al., 2011; Leibson et al., 2012; Kayani et al., 2009; Rochette et al., 2009; Vangel et al., 2005). Most recent, a study from 2004 to 2009 found that median hospital costs for adults were $13,000 for the 18-64 age group and $9,000 for the 65 and older age group. (Hu et al., 2013) In a 2005 study, unadjusted total mean one year costs were $77,872 for those aged 55-64 years, $76,903 for those aged 65-74 years and $72,733 for those aged 75-84 years in 2005 dollars (Thompson et al., 2012). In a study of the managed care population, initial hospitalization charges ranged from $32,627 for TBI alone to $103,667 for TBI along with other trauma (Davis et al., 2007). Another study found mean cost of treating a TBI to be $96,612, with inpatient rehabilitation accounting for on average $43,212, not including physician charges (Thompson, 2001).

Costs are known to vary due to severity. One case controlled found total health services ranged from $12,990 for mild TBI vs. $42,4441 for moderate/severe in 2009 dollars (Rockhill et al., 2011). As a comparison, total health services costs for non-TBI matched controls were $7,377 (Rockhill et al., 2011). Mean costs were 76% higher in the three years after injury for the mild TBI group and 5.75 times greater for the moderate/severe group compared to controls (Rockhill et al., 2011). Another case control study for that for definite and probable TBI, most incremental costs occurred within the first six months while significant long-term incremental medical costs were not apparent among one-year survivors (Leibson et al., 2012). Cost differences between possible TBI cases and matched controls were not as great in the first 6 months but were substantial among one-year survivors (Leibson et al., 2012).

Studies also found significant variation in cost by geographic region, type of service, and comorbidity. One study found the highest costs occurring in the states of California and Washington (Hu et al., 2013). State specific studies found varying costs but often categorized costs differently, making comparisons difficult (Kayani et al., 2009; Rochette et al., 2009; Vangel et al., 2005). A study specific to the geriatric population found hospitalization and inpatient rehabilitation costs significantly lower in the 75-84 age category while outpatient care costs and nursing home costs were lower in the younger age categories (Thompson et al., 2012). In a study of the Medicaid population in four states, the presence of co-morbid mental illness increased the cost of care for those with TBI (Wei et al., 2005). Total expenditures ranged by state from $6,093 to $10,907 for those without SMI, compared to $8,723 to $21,924 for those with SMI (Wei et al., 2005). Another study showed presence of psychiatric illness was associated with more than doubling of total costs for both inpatient and outpatient non-mental health care (Rockhill et al., 2011).

Finally, a national study of US Operation Enduring Freedom/Operation Iraqi Freedom (OEF/OIF) veterans treated in the VA found that OEF/OIF veterans with TBI had 4 times higher median health services costs relative to OEF/OIF veterans without TBI ($5,831 vs. $1,547) (Taylor et al., 2012). Mental health co-morbidities in the military population increase health services costs associated with TBI, similarly to civilian populations, with those with TBI and PTSD having a median cost of $5,053 relative to those with TBI alone being $2,391 (Taylor et al., 2012).

### 4.2 International Health Services Costs Associated With TBI

Health services costs associated with TBI have also been estimated for other countries. In China, hospitalization costs were found to be highest for traffic accidents and lowest for blows to the head (Yuan et al., 2012). Multiple factors were associated with higher acute hospitalization costs with doubling of length of stay associated with a 61% higher hospital cost (Yuan et al., 2012). The latest estimate for the cost of TBI in Europe, based on data from 30 European countries in 2010 was €2,697 in direct health care costs (Olsen et al., 2012). A study of England and Wales found that length of stay in critical care accounted for 51% of mean total costs, regular ward 38% and travel costs 5%. (Morris et al., 2008) Those aged 45-64 years had the highest costs. (Morris et al., 2008) In another European study based on 2004 data, average inpatient cost for TBI in Germany was €2,529, compared with €2,833 in Spain and €3,024 in Sweden. Average inpatient cost for concussion was €1,071 for Germany, €987 for Spain, and €927 for Sweden. Average inpatient cost for severe brain injury was €6,647 for Germany, €6,362 for Spain and €6,045 for Sweden (Berg, 2004).

### 4.3 Health Services Utilization Associated With TBI in the United States

TBI injuries are associated with approximately 275,000 hospitalizations and 1,365 million ED visits in the US annually (Faul, 2010). One study found that 81% used medical and allied health services, 66% used transport and 40% used vocational rehabilitation (Hodgkinson et al., 2000). In Georgia, during the first three months of post rehabilitation discharges, at least 80% saw an MD, 42% reported four or more MD visits, over 50% attended day rehabilitation programs, 42% had physical therapy, 36% occupational therapy, 33% speech pathology, and 11% had psychological counseling. (Philips et al., 2004) Re-hospitalization rates declined between the one and five year follow up period from 23% to 17% (Marwitz et al., 2001). While during the first year, orthopedic and reconstructive surgery were the primary reason (25%), infections accounted for 10% of readmissions at 1 year follow-up and 8% at 5 years after injury (Philips et al., 2004). Incidence of rehospitalizations relating to seizures ad psychiatric disorders were 12% at one year and rose to 19% at 5 years (Philips et al., 2004).

Financial incentives may influence end of life care for severe TBI (Holloway et al., 2010). Hospitals have been found to have a strong incentive and insurers a strong disincentive to pay for performing tracheotomies since doing so quintuples a hospital’s DRG associated reimbursement rate (Holloway et al., 2010). If a tracheotomy is not performed, the hospital has a financial incentive to discontinue aggressive treatment due to the lower fixed DRG reimbursement rate (Holloway et al., 2010). In another sample of 273 patients with TBI, only 26% were found to have received a neuropsychology evaluation. (NPE) (Schatz et al., 2001). These patients were younger, more likely to be involved in liability claims, achieved a higher functional rehabilitation and attended multiple rehabilitation facilities (Schatz et al., 2001).

A number of patient characteristics are significantly associated with utilization of various services. In a national study of the ED population diagnosed with isolated mild TBI, 44.3% were found to have received CT, 23.9% other non-extremity, non-chest x-rays, 17.1% wound care and 14.1% IV fluids (Bazarian et al., 2005). However, only 43.8% of the ED population were assessed for pain and of those with documented pain, only 45.5% received analgesics while in the ED (Bazarian et al., 2005). Almost 38% of the ED population was discharged from the ED without recommendations for follow-up (Bazarian et al., 2005). State specific studies again focused on different categories, thus are difficult to compare. In an Ohio study, the odds of requiring a ventilator (OR=3.66) and being admitted to the ICU (OR=2.51) were significantly higher for a TBI roadway injury when compared to non-TBI roadway injury (Rochette et al., 2009). In Michigan, more than 4/5 of study subjects received a CT scan while MRI and electroencephalography were used less frequently (Vangel et al., 2005). In Michigan, the most frequent prescriptions filled were anticonvulsant/mood stabilizers (Vangel et al., 2005), however, nationally the most frequently used medicines were analgesics and anxiolytics as well as anticonvulsants in more severe injuries (McGarry et al., 2002).

### 4.4 International Health Services Utilization Associated With TBI

Few studies were found in this review addressing utilization outside the United States. A study of TBI rehabilitation in Italy found that the mean LOS was 87.31, and 40.4% of patients had access to rehabilitation facilities after a month (Zampolini et al., 2011). A study of CT use and TBI in some European countries found that CT use in Spain was much lower than Sweden and Germany with only 6% of patients receiving a CT (Berg, 2004).

### 4.5 Disparities in Cost and Utilization Associated With TBI in the United States

Disparities in health services cost and utilization have been found in the US based on race. Non-White race and lack of insurance were found to be associated with lower likelihood of placement for rehabilitation post-discharge (Heffernan et al., 2011). In South Carolina, uninsured Black females had a lower likelihood of hospitalization (Selassie et al., 2004). A national study of the ED population diagnosed with mild TBI found that Hispanics were more likely to receive a nasogastric tube, while non-Whites were more likely to receive care by a resident and less likely to be sent back to a referring physician after discharge (Bazarian et al., 2003). American Indians and Alaskan Natives (AI/AN) had the highest age adjusted rates of hospitalization for TBI relative to other race/ethnicities (Rutland-Brown et al., 2005)

In one study, private insurance was associated with a shorter length of stay (Heffernan et al., 2011). However, in another study insured patients had a longer length of stay in the intensive care unit (ICU), and lower mortality (Alban et al., 2010). Additionally, in a national study mean and median acute care charges were only slightly higher for males compared with females, however mean and median charges were highest for the western region of the US and for persons covered by Medicaid and those treated in urban teaching hospitals (Schootman et al., 2003).

In a study of unmet needs of individuals with TBI, the most prevalent unmet needs were for memory (50.5%), job skills (46.3%), and increasing income (50.5%) (Heinemann et al., 2002). Black, younger and single individuals as well as those dependent in one or more daily activities and with more recent injuries had greater unmet needs (Heinemann et al., 2002). A review of TBI Medicaid Waivers in six states showed that the ability to obtain a waiver was associated with the social-medical-political climate, ability to strengthen access and reduce barriers, and expenditure of resources (Spearman et al., 2001).

### 4.6 International Disparities in Cost and Utilization Associated With TBI

Among 79 patients with moderate/severe TBI in Netherlands, those with a high locus of control were more likely to visit medical specialists and use supportive care than those with lower levels of internal locus of control. (Willemse-van, 2009). In Australia, rural TBI patients were found to be more likely treated in a non-inpatient setting than urban TBI patients, though this did not appear to significantly impact functional outcomes (O’Callaghan et al., 2009).. Finally, terror victims with TBI were found to have higher rates of brain surgery but no difference in length of stay relative to non-terror victims in Israel (Schwartz et al., 2008).

### 4.7 Conclusions

We reviewed a decade of literature regarding the utilization and cost of health services for TBI in civilian and military adults in the US as well as other countries. We found that cost estimates vary depending on the study population, TBI severity and presence of other mental health co-morbidities. We also found there was evidence of significant racial/ethnic disparities in utilization. However, there is need for further research on cost of TBI over time and better understanding of racial/ethnic, geographic and socioeconomic variations in cost and utilization among individuals with TBI in both civilian and military/Veteran populations in the US and worldwide.

Our study has a number of limitations. We only searched literature since the year 2000 and included studies only published in the English language. We also only reviewed published studies, which may reflect publication bias, as some analyses are not published.

We can conclude, however, that the cost and utilization of TBI is an important area of research for the next 5 years, especially considering the growing recognition of the impact of mental health on health costs and outcomes. A potential concern, which has been addressed only marginally in the literature, is racial/ethnic, geographic, and socio-economic disparities in utilization of health services for TBI and its associated co-morbid conditions. There is also a need to control for severity and follow patients over time to truly compare costs and utilization. Future research should investigate the change in cost during the first year vs. over the lifetime, and costs for those who receive mental health services vs. those who do not.
